# Supplementary material for: Individual differences in honey bee behavior enabled by plasticity in brain gene regulatory networks
Source: eLife. 2020 Dec 22;9:e62850. doi: 10.7554/eLife.62850 (PMC7755388; doi:10.7554/eLife.62850)
Supplement: Transparent reporting form [file elife-62850-transrepform.docx]

***eLife’s* transparent reporting form**

We encourage authors to provide detailed information *within their submission* to facilitate the interpretation and replication of experiments. Authors can upload supporting documentation to indicate the use of appropriate reporting guidelines for health-related research (see [EQUATOR Network](http://www.equator-network.org/%20)), life science research (see the [BioSharing Information Resource](https://biosharing.org/)), or the [ARRIVE guidelines](http://www.plosbiology.org/article/info:doi/10.1371/journal.pbio.1000412) for reporting work involving animal research. Where applicable, authors should refer to any relevant reporting standards documents in this form.

If you have any questions, please consult our Journal Policies and/or contact us: [editorial@elifesciences.org](mailto:editorial@elifesciences.org).

**Sample-size estimation**

- You should state whether an appropriate sample size was computed when the study was being designed
- You should state the statistical method of sample size computation and any required assumptions
- If no explicit power analysis was used, you should describe how you decided what sample (replicate) size (number) to use

Please outline where this information can be found within the submission (e.g., sections or figure legends), or explain why this information doesn’t apply to your submission:

Although the behavioral groups of interest were identified in a previous study (Naeger et al. 2013, Current Biology), we lacked knowledge about their frequency. We therefore calculated the maximum number of colonies that we could track given constraints on field season length and tracking equipment capacity and achieved that number. For RNAseq and ATACseq, we obtained the maximum number of individuals per colony from the behavioral groups of interest that were alive at the end of tracking. These numbers are within the range typically used and published in other similar studies, and above the minimum number of biological replicates necessary for statistical analysis of differential brain gene expression and chromatin accessibility; no explicit power analysis was used. The statistical approaches used are well established for similar datasets.

**Replicates**

- You should report how often each experiment was performed
- You should include a definition of biological versus technical replication
- The data obtained should be provided and sufficient information should be provided to indicate the number of independent biological and/or technical replicates
- If you encountered any outliers, you should describe how these were handled
- Criteria for exclusion/inclusion of data should be clearly stated
- High-throughput sequence data should be uploaded before submission, with a private link for reviewers provided (these are available from both GEO and ArrayExpress)

Please outline where this information can be found within the submission (e.g., sections or figure legends), or explain why this information doesn’t apply to your submission:

Behavioral experiments were performed on 6 colonies, with all (800) individuals per colony monitored continuously (line 123, Figure 1 legend). RNAseq and ATACseq were performed on brains of individuals from two unrelated colonies (Figure 2, line 667, Supplemental File 2) for a total of 45 individuals across groups and colonies (detailed sample information given in Supplemental File 2, numbers of individuals per group from each colony also visible in Figure 2). All individuals were used for statistical analysis (i.e., no outliers discovered nor removed). As usual for these kinds of data, genes and chromatin peaks with low counts (less than one count per million in at least two samples) were removed prior to analysis (lines 745-747; lines 767-769). Sequence data are available in the NCBI Short Read Archive, accession PRJNA593999 (line 933).

**Statistical reporting**

- Statistical analysis methods should be described and justified
- Raw data should be presented in figures whenever informative to do so (typically when N per group is less than 10)
- For each experiment, you should identify the statistical tests used, exact values of N, definitions of center, methods of multiple test correction, and dispersion and precision measures (e.g., mean, median, SD, SEM, confidence intervals; and, for the major substantive results, a measure of effect size (e.g., Pearson's r, Cohen's d)
- Report exact p-values wherever possible alongside the summary statistics and 95% confidence intervals. These should be reported for all key questions and not only when the p-value is less than 0.05.

Please outline where this information can be found within the submission (e.g., sections or figure legends), or explain why this information doesn’t apply to your submission:

Statistical analyses are described in the Methods section (lines 737-773, 804-859, 868-907), with references to software or other papers as appropriate. Raw data for behavioral counts per individual are provided in Supplemental File 1. Results for individuals are shown where appropriate (e.g., Figure 2, Figure 4, and Figure 4- figure supplement 1). Multiple testing corrections were done for all analyses of gene expression, chromatin accessibility, and Gene Ontology functional enrichment (lines 197, 204, 213, 223, 751, 773), tests of transcription factor enrichment (lines 269, 271, 852-854), and gene regulatory network analyses (lines 882-883). For hypergeometric tests of overlap, representation factors (RF) are given as a measure of effect size (amount of overlap relative to random expectation) along with exact p-values (also given in Supplemental File 9). Correlation coefficients are given for correlations of both gene expression and chromatin accessibility principal components with behavioral specialist score. Further details, including gene lists, PC loadings, uncorrected and corrected p-values for gene ontology enrichment, motif enrichment, and hypergeometric tests are provided in Supplemental Files 1, 3, and 9.

(For large datasets, or papers with a very large number of statistical tests, you may upload a single table file with tests, Ns, etc., with reference to sections in the manuscript.)

**Group allocation**

- Indicate how samples were allocated into experimental groups (in the case of clinical studies, please specify allocation to treatment method); if randomization was used, please also state if restricted randomization was applied
- Indicate if masking was used during group allocation, data collection and/or data analysis

Please outline where this information can be found within the submission (e.g., sections or figure legends), or explain why this information doesn’t apply to your submission:

Samples were assigned to groups for data analysis based upon behavior as determined by our automatic behavioral tracking system and behavioral specialist and generalist scores (described in the following sections: “Behavioral tracking,” “Egg-laying detector,” “Filtering and annotation of entrance data,” and “Specialist and generalist scores”). The behavior of all individuals was analyzed with the same egg-laying and foraging detectors, and all individuals were assigned specialist and generalist scores (provided in Supplemental File 1). Choice of individuals for sequencing is explained in section “Selection of bees for sequencing.”

**Additional data files (“source data”)**

- We encourage you to upload relevant additional data files, such as numerical data that are represented as a graph in a figure, or as a summary table
- Where provided, these should be in the most useful format, and they can be uploaded as “Source data” files linked to a main figure or table
- Include model definition files including the full list of parameters used
- Include code used for data analysis (e.g., R, MatLab)
- Avoid stating that data files are “available upon request”

Please indicate the figures or tables for which source data files have been provided:

Figure 1: details of experimental setup for tracked colonies (e.g., dates recorded) are provided in Supplemental File 10

Figure 2: generalist and specialist scores are given in Supplemental File 1

Figure 2: detailed information on individuals selected for sequencing is provided in Supplemental File 2; lists of differentially expressed genes and differentially accessible chromatin peaks are provided in Supplemental Files 3 and 4

Figure 3: transcription factor motif enrichment results are provided in Supplemental File 7

Figure 4: predicted gene regulatory networks (GRN), GRN module correlations with behavior and physiological measurements, and importance scores of transcription factors from class prediction analysis are provided in Supplemental File 8
